# Balancing mTOR Signaling and Autophagy in the Treatment of Parkinson’s Disease

**DOI:** 10.3390/ijms20030728

**Published:** 2019-02-08

**Authors:** Zhou Zhu, Chuanbin Yang, Ashok Iyaswamy, Senthilkumar Krishnamoorthi, Sravan Gopalkrishnashetty Sreenivasmurthy, Jia Liu, Ziying Wang, Benjamin Chun-Kit Tong, Juxian Song, Jiahong Lu, King-Ho Cheung, Min Li

**Affiliations:** 1 Mr. and Mrs. Ko Chi Ming Centre for Parkinson’s Disease Research, School of Chinese Medicine, Hong Kong Baptist University, Hong Kong SAR 999077, China; zzhou1022@gmail.com (Z.Z.); nkyangchb@gmail.com (C.Y.); ashokenviro@gmail.com (A.I.); senthilnslab@gmail.com (S.K.); sravangs@gmail.com (S.G.S.); liujiatheone@hotmail.com (J.L.); wangziying.12@163.com (Z.W.); benjamintck@gmail.com (B.C.-K.T.); kingho@hkbu.edu.hk (K.-H.C.); 2 Medical College of Acupuncture-Moxibustion and Rehabilitation, Guangzhou University of Chinese Medicine, Guangzhou 510006, China; juxian.song@gmail.com; 3 State Key Laboratory of Quality Research in Chinese Medicine, Institute of Chinese Medical Sciences, University of Macau, Taipa, Macau SAR 999078, China; jiahonglu@um.edu.mo

**Keywords:** mTOR, autophagy, Parkinson’s disease

## Abstract

The mammalian target of rapamycin (mTOR) signaling pathway plays a critical role in regulating cell growth, proliferation, and life span. mTOR signaling is a central regulator of autophagy by modulating multiple aspects of the autophagy process, such as initiation, process, and termination through controlling the activity of the unc51-like kinase 1 (ULK1) complex and vacuolar protein sorting 34 (VPS34) complex, and the intracellular distribution of TFEB/TFE3 and proto-lysosome tubule reformation. Parkinson’s disease (PD) is a serious, common neurodegenerative disease characterized by dopaminergic neuron loss in the substantia nigra pars compacta (SNpc) and the accumulation of Lewy bodies. An increasing amount of evidence indicates that mTOR and autophagy are critical for the pathogenesis of PD. In this review, we will summarize recent advances regarding the roles of mTOR and autophagy in PD pathogenesis and treatment. Further characterizing the dysregulation of mTOR pathway and the clinical translation of mTOR modulators in PD may offer exciting new avenues for future drug development.

## 1. Introduction of mTOR

The target of rapamycin (TOR) was first identified as a target protein of rapamycin, encoded by TOR1 and TOR2 alleles, through screening of rapamycin-resistant mutant yeast [1]. This study showed rapamycin could form a complex with FK506-binding protein (FKBP), leading to cell cycle arrest in the G1 phase, which is mediated by TOR1 and TOR2 [1]. Subsequent studies of mammalian cells found homologous proteins, termed mammalian targets of rapamycin (mTOR), which shared more than 40% consistency in amino acid sequence with yeast TOR1 and TOR2 [2]. In addition, the function of mTOR was also related to the rapamycin-FKBP12 induced cell cycle arrest [2,3].

**Figure 1:**
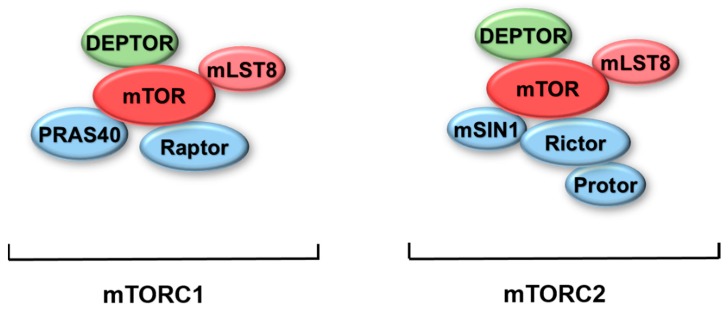
Protein components of mTORC1 and mTORC2. Both mTORC1 and mTORC2 include the same macromolecules such as mTOR, mLST8, and DEPTOR. Apart from these components, mTORC1 also contains PRAS40 and Raptor. Correspondingly, mTORC2 contains mSIN1, Rictor, and Protor. Abbreviation: mTORC1, mTOR complex 1; mTORC2, mTOR complex 2; mTOR, Mammalian targets of rapamycin; mLST8, Mammalian lethal with sec-13 protein 8; DEPTOR, DEP-domain containing mTOR-interacting protein; PRAS40, Proline-rich Akt substrate of 40 kDa; Raptor, Regulatory associated protein of mammalian target of rapamycin; mSIN1, Mammalian stress-activated map kinase-interacting protein 1, Rictor, Rapamycin-insensitive companion of mTOR; Protor, Protein observed with Rictor.

mTOR, which is also termed as FKBP12-rapamycin complex-associated protein (FRAP), is a conserved serine/threonine protein kinase, and mTOR belongs to the phosphoinositide-3-kinase (PI3K)-related kinase family of protein kinases [4]. mTOR constitutes the catalytic component of two distinct multiprotein complexes: mTOR complex 1 (mTORC1) and mTOR complex 2 (mTORC2) (Figure 1) [5].

mTORC1 contains mTOR, mammalian lethal with SEC13 protein 8 (mLST8), DEP (DVL, Egl-10, pleckstrin)-domain containing mTOR-interacting protein (DEPTOR), proline-rich Akt substrate of 40 kD (PRAS40), and regulatory associated protein of mammalian target of rapamycin (Raptor) (Figure 1) [6,7,8,9,10]. In this complex, mTOR-combined DEPTOR and PRAS40 can negatively regulate mTORC1 activity [6,10]. mTORC1 plays a key role in regulating cell growth, cell size, and proliferation [8,11,12]. mTORC1 is a core component in a series of signaling networks. Additionally, it senses different stimuli such as insulin level, energy level, and amino acid level, and is involved in protein synthesis, lipid metabolism, glycolytic metabolism, and autophagy [13,14].

mTORC2 consists of mTOR, mLST8, DEPTOR, rapamycin-insensitive companion of mTOR (Rictor), mammalian stress-activated map kinase-interacting protein 1 (mSIN1), and protein observed with Rictor (Protor) (Figure 1) [6,15,16,17,18,19]. mTORC2 also regulates many cellular processes, such as cell growth, proliferation, metabolism, and cell motility via the AGC kinase family member Akt, serum/glucocorticoid regulated kinase (SGK), protein kinase C (PKC), and filamin A [20,21,22,23]. The well-known substrate of mTORC2 is Akt. Akt can be fully activated when it is phosphorylated by 3-phosphoinositide dependent protein kinase-1 (PDK1) at Thr308 site, and subsequently phosphorylated by mTORC2 at Ser473 site [24,25]. Actually, Yang et al. found that mSIN1, a component of mTORC2, mediates a positive feedback loop between mTORC2 and Akt [26]. The phosphorylation of mSIN1 at Thr86 site, which is induced by phospho-Akt, enhances mTORC2 activity in response to growth factors [26,27].

The PI3K/Akt/mTOR signaling pathway has been extensively studied because it plays a crucial role in controlling cell growth, in maintaining cell viability, and in determining a cell’s life span [28,29]. Insulin receptor substrate (IRS) activates phosphatidylinositol 3-kinase (PI3K) upon presence of growth factors, recruiting PIP2 to the plasma membrane, and enhancing transformation of PIP2 to PIP3. PIP3 promotes the phosphorylation of Akt on the Thr308 and Ser473 sites by PDK1 and mTORC2, respectively. Once being fully activated, Akt phosphorylates and inhibits tuberous sclerosis complex (TSC), which is the negative regulator of Ras homolog enriched in brain (Rheb) and finally leads to the activation of mTORC1 [30]. There are two well-established downstream effectors being phosphorylated by mTORC1, p70 ribosomal S6 kinase (P70S6K) and eukaryotic initiation factor 4E (eIF4E) binding protein 1 (4EBP1); both of them are main regulators of cap-dependent protein synthesis [31,32].

## 2. Role of mTOR in Autophagy

Autophagy is an evolutionarily conserved turnover process that exerts great importance on the clearance of long-lived proteins, aggregated protein, or dysfunctional organelles, and provides energy and macromolecular precursors in return [33]. Autophagy has been widely divided into three sorts: macro-autophagy, micro-autophagy, and chaperone-mediated autophagy [34,35]. The term “autophagy” in this review refers to macro-autophagy. The process of autophagy includes initiation, nucleation, elongation, and formation of a double-membrane autophagosome, followed by the fusion of the autophagosome with a lysosome to form autolysosomes to degrade and recycle autophagosome-sequestered substrates [33]. It has been reported that mTOR plays a complex role in the induction, process, and termination of autophagy. Here, we will briefly summarize several key mTOR-related pathways that regulate autophagy activity.

### 2.1. mTOR/AMPK/ULK1 Signaling

**Figure 2:**
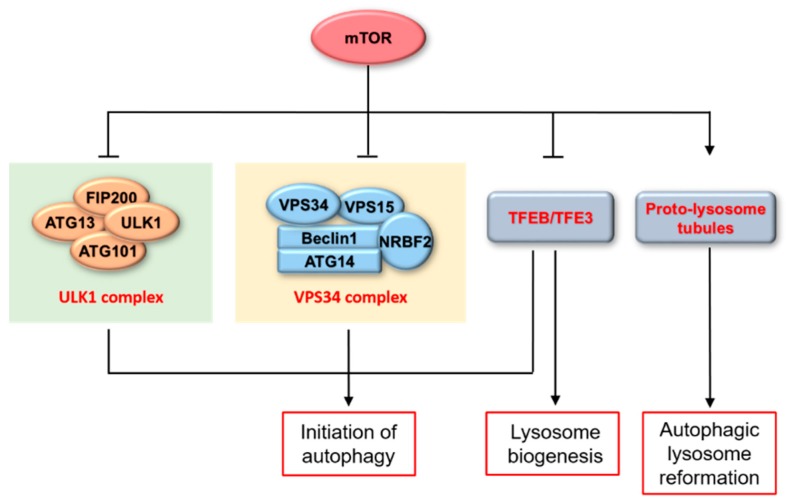
Role of mTOR in autophagy. mTOR plays a crucial role in the regulation of autophagy flux, including the formation of phagophore and autophagosome, the degradation of autolysosomes, and the reformation of autophagic lysosomes.

Unc51-like kinase 1 (ULK1) interacts with ATG13, ATG101 and focal adhesion kinase family interacting protein of 200 kD (FIP200), making up ULK1 complex (Figure 2) [36,37,38]. This complex is a critical initiator of autophagy, and its activity is mainly regulated by being phosphorylated at different sites by the combination of mTORC1 and AMP-activated protein kinase (AMPK) [39,40]. Normally, mTORC1 phosphorylates ULK1 on the P757 site and disrupts the interaction of AMPK and ULK1, inhibiting the initiation of autophagy [39]. Upon nutrient deprivation or other cellular stresses, ULK1 is released from mTORC1, which has been inhibited, and is activated through being phosphorylated by AMPK at multiple sites [39]. This phosphorylation by AMPK has been shown to induce autophagy in most cases [39,41]. ATG13, one component of ULK1 complex, also can be phosphorylated by activated mTOR, leading to the decreased activity of ULK1 complex and autophagy inhibition [42]. Thus, inhibition of mTORC1 induces ULK1 complex-mediated autophagy, which can be suppressed by inhibition or deficiency of ULK1 [43].

### 2.2. mTOR/VPS34-ATG14 Complex Signaling

Vacuolar protein sorting 34 (VPS34), also known as PIK3C3, is the catalytic subunit of type Ⅲ PI3K. VPS34 plays an important role in endosome trafficking and pre-autophagosome formation with the function of converting phosphatidylinositol (PI) to phosphatidylinositol 3-phosphate (PI3P) [44]. VPS34, VPS15, and beclin 1 constitute core subunits of two VPS34 complexes, complex Ⅰ and complex Ⅱ, with ATG14 and UVRAG separately [38]. Among them, Atg14-containing VPS34 complex is involved in autophagy induction, facilitating the formation of isolation membrane on the endoplasmic reticulum (ER) membrane (Figure 2) [45,46,47]. Although it has been reported that VPS34 activates P70S6K phosphorylation in mammalian cells in the presence of nutrients, it remains unclear whether VPS34 influences mTOR activation directly [48,49]. Meanwhile, it has been reported that mTORC1 inhibits the activity of the VPS34 complex by directly phosphorylating ATG14 on a series of sites [50]. Mutation of these sites, which is resistant to inhibition by mTOR, could enhance autophagy flux [50]. In recent years, nuclear receptor binding factor 2 (NRBF2) has been reported to act as the fifth subunit of the Atg14-containing VPS34 complex [51]. NRBF2 is indispensable for the integrity of this complex [51,52,53,54] and has been implicated in neurodegenerative diseases such as Alzheimer’s disease [55]. In addition, NRBF2 can be phosphorylated by mTORC1 at S113 and S120 and its dephosphorylated form enhances VPS34 complex assembly and activity, promoting autophagy flux [52].

### 2.3. mTOR/TFEB/TFE3

Both TFEB and TFE3 are members of the MiT-TFE family, belonging to helix-loop-helix leucine-zipper transcription factors [56,57]. TFEB is a master regulator of genes related to lysosomal biogenesis and autophagy, and recently TFE3 has also been found to regulate the transcription of genes that largely overlap with the ones regulated by TFEB [57,58]. The common mechanism underlying shuttling of transcription factors between nucleus and cytoplasm mainly depends on whether transcription factors are phosphorylated or not [57,58]. When nutrients are present, TFEB and TFE3 are recruited to the membranes of lysosomes and undergo mTOR-dependent phosphorylation, at S211 of TFEB and S321 of TFE3, creating a binding site for the chaperone 14-3-3 and thus sequestrating them in the cytosol [59]. Upon nutrient deprivation, together with mTOR inactivation, dephosphorylated TFEB and TFE3 translocate to the nucleus and induce lysosomal biogenesis and autophagy [59]. Additionally, TFEB nuclear export is induced by hierarchical phosphorylation of Ser142 and Ser138 by activated mTOR [60]. Thus, mTOR plays a critical role in both autophagic and lysosomal biogenesis through regulating TFEB and TFE3 nuclear-cytoplasmic shuttling.

### 2.4. mTOR in Autophagic Lysosome Reformation (ALR)

At the termination of autophagy, lysosomes are recycled from autolysosomes through a process termed ALR, which includes proto-lysosome tubules generation, elongation, and scission [61]. This process exerts great importance throughout autophagy. Inhibition of ALR increases cells’ sensitivity to starvation and, over the long term, leads to death. [62]. During short-term food deprivation, mTOR is inhibited; while during long-term starvation, it is reactivated. This reactivation is essential for proto-lyosome tubule reformation [63]. What is more, mTOR is also initially inactivated and then reactivated in H_2_O_2_-induced autophagy, mediating the process ALR to regenerate functional lysosomes [64]. It has been found that mTOR inhibits VPS34 complex activity through phosphorylating UVRAG on Ser550 and Ser571 sites. It thereby reduces PI3P production, resulting in an increase in number and length of proto-lysosome tubules, due to impairment of tubule scission, and indicating the indispensable function of mTOR in the scission of proto-lysosome tubules [62].

## 3. Role of mTOR in Parkinson’s Disease

Parkinson’s disease (PD) is one of the most common neurodegenerative diseases in the world, characterized mainly by dopaminergic neuron loss in the substantia nigra pars compacta (SNpc) and the accumulation of α-synuclein-containing inclusions, named Lewy bodies. Genetic mutations are the leading cause of the disease, but it can also be caused by aging or dopaminergic neuron-specific toxins, such as 6-hydroxydopamine (6-OHDA), 1-methyl-4-phenyl-1,2,3,6 tetrahydropyridine (MPTP), and rotenone [65]. Among these toxins, MPTP has been widely used for developing the PD animal model [66]. Actually, MPTP itself is nontoxic and can penetrate the blood–brain barrier. While in the brain, it can be oxidized to MPP^+^, which is toxic to dopaminergic neurons [67]. Thus, MPTP is usually used for animal models of PD, and MPP^+^ is used for cell models of PD. As mTOR signaling is a central hub of signaling networks in cells, it has been widely explored and has been found to have a complex relationship with PD. Both activation and inactivation of mTOR signaling are involved in the different stages of PD.

α-synuclein accumulation is a hallmark of PD, which has been implicated in the pathogenesis of sporadic and familial PD [68,69]. mTOR protein expression levels were increased in the temporal cortex of patients displaying α-synuclein accumulation [70]. Additionally, upon overexpression of α-synuclein, it can inhibit autophagy possibly through inducing mTOR activity and mimic the symptoms of PD [71]. Conversely, rapamycin, an inhibitor of mTOR, can restore the increased mTOR activity caused by α-synuclein overexpression [71]. What is more, A53T α-synuclein, a common mutation of α-synuclein in PD, upregulates mTOR/P70S6K signaling and impairs autophagy, contributing to the aggregation of toxic A53T α-synuclein [72]. On the other hand, depletion of mTOR results in the induction of autophagy, leading to clearance of A53T α-synuclein [72]. These findings indicate that mTOR activities are increased in PD and α-synuclein accumulation may contribute to this process.

RTP801/REDD1 is a stress-related protein, whose expression is markedly elevated in neurons of the SNpc in PD patients compared to control patients [73]. RTP801 interacts with TSC2, inhibiting activation of mTOR and thus leading to neuron cell death; this process may account for the neuron loss in the SNpc of PD patients [74,75]. An increase in RTP801 expression is also observed in cellular models of PD (6-OHDA, MPP^+^ or rotenone) and in animal models of PD. In both cases, the increased RTP801 expression is accompanied by decreased mTOR activity [73].

It is well known that mTOR signaling is of great importance in cell proliferation and survival. The phosphorylation of Akt, the upstream kinase of mTOR, is decreased in the MPP^+^-induced cellular model of PD, attenuating the activation of mTOR [76]. In addition, AMPK is a negative regulator of mTOR, which is activated in different cellular models of PD [77]. Thus, in PD models induced by toxins, both increased Akt and AMPK could negatively regulate the activity of mTOR, leading to the impairment of downstream 4EBP1 and P70S6K-related protein synthesis. This protein synthesis is essential for cell long-term survival. Furthermore, neuronal cell death induced by PD toxins can be partially restored via overexpression of functional mTOR [77].

## 4. Potential PD Treatment by Targeting mTOR

Since an increase in toxic protein aggregation and a loss of dopaminergic neurons are the symptoms of PD, symptomatic treatment and prevention of neuron death are the primary strategies in the therapy to manage features and progress of PD.

### 4.1. Treatment of PD by Combining L-DOPA with mTOR Inhibitors

**Figure 3:**
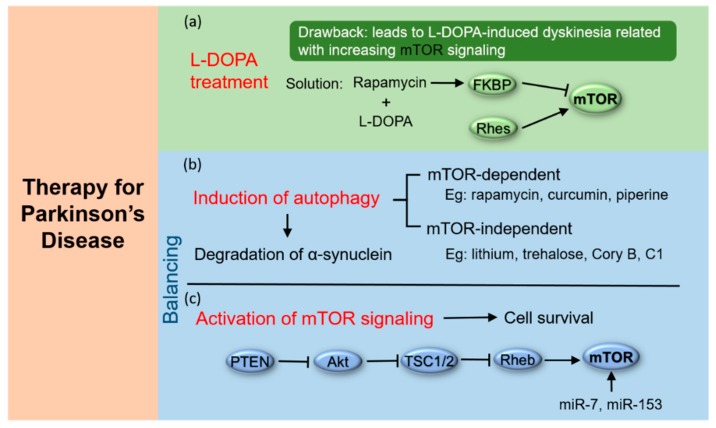
Potential for using mTOR in PD treatment. (**a**) Inhibition of mTOR signaling, through pharmacological blockade of mTOR or reduction of Rhes, provides a better stage for the L-DOPA therapy of PD. (**b**) The induction of autophagy by either mTOR-dependent or -independent pathway, enhances the degradation of toxic α-synuclein to alleviate the symptoms of PD. (**c**) Activation of Akt or Rheb, specific ablation of PTEN or overexpression of miR-7 and miR-153 could increase mTOR signaling to prevent neuron cell death. Furthermore, a balance between activation of mTOR signaling and enhancement of autophagy needs to be accurately managed in the treatment of PD. Abbreviations: PD, Parkinson's disease; Rhes, Ras homolog enriched in striatum; Rheb, Ras homolog enriched in brain; miR, MicroRNA.

The dopamine precursor drug, L-DOPA has been clinically used for the initial treatment of PD for more than 50 years [78]. L-DOPA compensates for reduced dopamine levels caused by the loss of dopaminergic neurons. However, with long-term L-DOPA treatment, most patients start to experience motor response fluctuations or dyskinesia [79,80]. By using a genetic association approach, Martin-Flores et al. have detected genetic variability in the mTOR pathway and found it involved in the development of L-DOPA-induced dyskinesia [81]. Persistent activation of mTOR signaling in the striatum has been found in L-DOPA-induced dyskinesia [82]. L-DOPA induces increased dopamine D1 receptor-mediated phosphorylation of mTOR downstream substrates, P70S6K and 4EBP1, indicating enhanced activity of mTOR signaling in medium spiny neurons; this increased mTOR signaling activity correlates positively with L-DOPA-induced dyskinesia [82]. Thus, inhibition of mTOR activity may function in the reduction of dyskinesia caused by L-DOPA. mTOR inhibitor rapamycin has been used on animal model of PD in combination with L-DOPA when it successfully prevents increased activity of mTOR and reduces dyskinesia produced by L-DOPA [82]. Similarly, depletion of Ras homolog enriched in striatum (Rhes) also reduces mTOR signaling and diminishes L-DOPA-induced dyskinesia [83]. Rhes is a highly enriched striatal-specific protein, which binds to and activates mTOR in the striatum [83]. Thus, reducing or depleting Rhes is another way to limit the activation of mTOR in the development of L-DOPA-induced dyskinesia. Taken together, as shown in Figure 3, inhibition of mTOR signaling, through pharmacological blockade of mTOR or reduction of Rhes, provides a beneficial effect on the L-DOPA therapy of PD [84].

### 4.2. Induction of Autophagy

Autophagy dysfunction has been reported to be associated with the pathogenesis of many neurodegenerative diseases including PD [85]. Genetic studies have identified mutations in genes which encode for components of the autophagy–lysosome pathway, including α-synuclein, leucine-rich repeat kinase 2 (LRRK2), glucosidase beta acid 1 (GBA1), scavenger receptor class B member 2 (SCARB2), Parkin, PTEN-induced putative kinase (PINK1), DJ-1, Fbxo7, and vacuolar protein sorting 35 (VPS35), and these mutations are associated with increasing risks for developing PD [86,87,88]. Pathological studies have observed decreased expression of autophagy–lysosome pathway-related proteins levels and lysosomal enzyme activity in PD patients [87]. For instance, lysosome depletion was indicated by decreased levels of LAMP1 in the SNpc of PD patients. Meanwhile, negative regulation of lysosomal enzymes, like GCase, have been demonstrated in different brain regions and cerebrospinal fluid of PD patients [89]. Importantly, TFEB expression in the nuclear compartment of dopaminergic neurons was significantly decreased in the postmortem SNpc of PD patients compared to controls, indicating that the subcellular localization of TFEB was changed [90]. Moreover, TFEB co-localized with Lewy bodies in the same region [90]. Given the fact that autophagy impairment is implicated in the pathogenesis of PD, autophagy is a key to the degradation of α-synuclein. It is proposed that autophagy-enhancing strategies have great potential as disease-modifying therapies for PD [91]. Indeed, genetic manipulations (such as TFEB or Beclin 1 overexpression) could enhance autophagy, thereby protecting nigral neurons from α-synuclein toxicity in PD animal models [90]. Similarly, rapamycin has been well studied for the treatment of PD in animal models; it has been found to enhance autophagy flux and degrade neurotoxic proteins partially by inhibiting mTOR, thereby boosting lysosome biogenesis and autophagosome formation [92]. In addition to enhancing degradation of aggregate-prone proteins in PD models with activated autophagy, rapamycin blocks the translation of RTP801 by selectively inhibiting actions of mTOR, restoring the mTORC2-dependent phosphorylation of Akt, and maintaining cellular metabolism [93,94]. By inhibiting RTP801 activity and stimulating autophagy, rapamycin exerts neuroprotective influence on animal models of PD induced by 6-OHDA and MPTP [93,94]. In addition, several small molecules have been reported to induce mTOR-dependent autophagy and enhance the degradation of A53T α-synuclein in neuron cells; the latter is toxic and known to accelerate the development of PD symptoms [72]. For example, curcumin, culinary spice, plays a neuroprotective role in an A53T α-synuclein cell model of PD by enhancing autophagic degradation of A53T α-synuclein via inhibiting mTOR/P70S6K signaling [72]. Piperine, an alkaloid that gives black pepper its pungency, inhibits mTOR via activation of PP2A and then induces autophagy, thereby rescuing neurons (whether in cell culture or in mice) from rotenone neurotoxicity [95].

However, mTOR-dependent autophagy enhancers may compromise cell growth because mTOR signaling is such a significant signaling hub, modulating both cell proliferation and survival. Moreover, mTOR is essential for cellular functions including synaptic plasticity, memory formation and retention [96,97]. Thus, in order to avoid the negative effects of mTOR inactivation, small molecules that enhance the activity of autophagy independent of mTOR inhibition may be advantageous for PD treatment. In vitro studies, including our studies, have demonstrated that several compounds, such as lithium [98], trehalose [99], Corynoxine B [100], and a synthesized curcumin derivative termed C1 [101], can activate autophagy independent of mTOR, still leading to enhanced degradation of α-synuclein associated with PD. It has been reported that autophagy can be boosted by lowering intracellular inositol 1,4,5-trisphosphate (IP3) level independent of mTOR signaling [98]. Sarkar et al. have found that lithium induces autophagy through inhibiting activity of inositol monophosphatase (IMPase), which is essential in the regulation of intracellular free inositol and IP3 levels [98]. This induction of autophagy by lithium contributes to the clearance of mutant α-synuclein in stable inducible PC12 cells via decreasing IP3 levels, but rapamycin does not affect IP3 levels. They also reported that induction of autophagy by combination of mTOR-dependent and -independent pathways has an additive effect on the clearance of mutant α-synuclein in PC12 cells by using both rapamycin and lithium [98,102]. Overall, induction of autophagy in a mTOR-dependent or -independent manner may serve as a promising therapeutic target to degrade α-synuclein in PD treatment.

### 4.3. Activation of mTOR Signaling

The hallmark of PD is the loss of dopaminergic neurons in the SNpc, accompanied by decreased levels of dopamine, making it important to prevent dopaminergic neuron death [103]. Since mTOR signaling is a key regulator of protein synthesis, cell proliferation and survival, and mTOR inhibition leads to progressive neuron degeneration and a PD-like phenotype, it is necessary to retain the activity of mTOR signaling for its protective role in neurons [104]. Activation of mTOR requires a GTP-charged form of Rheb [105]. And TSC1/2 is an upstream negative regulator of mTOR, which has GTPase-activating protein (GAP) activity for Rheb [106,107]. TSC1/2 is a downstream target negatively regulated by Akt-mediated phosphorylation [30,108]. The connection between these proteins establishes a wide stage for stimulation of mTOR signaling. For example, viral vector transduction of dopaminergic neurons with Akt or Rheb activates mTOR signaling and restores the neurons’ ability to regenerate axons; this regenerative ability has valuable implications for the treatment of PD [109]. Moreover, the specific ablation of PTEN, an upstream negative regulator of Akt, contributes to activation of mTOR signaling and is neuroprotective in mouse models of PD [104].

MicroRNAs (miRs) are a class of small RNA molecules that play an essential role in the post-transcriptional regulation of gene expression via translational repression and mRNA degradation [110,111]. Recent studies have found that two miRs, miR-7, and miR-153, mainly expressed in neurons, negatively regulate α-synuclein expression [112]. In primary cortical neurons, overexpression of miR-7 and miR-153 promotes the mTOR/p70S6K signaling cascade and attenuates MPP^+^-induced neurotoxicity, although the underlying mechanism remains elusive [113]. Thus, overexpression of miRs by viral transduction, thereby inhibiting neuron cell death, may provide another potential approach in the therapy of PD patients. Taken together, activation of mTOR to restore neuronal survival may serve as a promising therapeutic strategy for PD treatment.

## 5. Conclusions

mTOR plays an important role in regulating neuronal functions and autophagy. Given the importance of mTOR, targeting mTOR is a potentially effective therapeutic target for PD (Figure 3). In terms of therapy of PD, it is crucial to accelerate the clearance of aggregated toxic proteins in neurons. Autophagy is a key pathway for promoting degradation of α-synuclein; thus, enhancing autophagy flux seems to be an effective way for PD treatment. As the central role of mTOR in autophagy regulation, mTOR-dependent autophagy enhancers hold great promise for PD treatment. As such, on the one hand, inhibiting mTOR signaling appears to be a viable treatment strategy. On the other hand, maintaining a certain level of mTOR activity is necessary because mTOR signaling is essential for cellular survival and growth. Importantly, mTOR regulates multiple essential cellular functions including synaptic plasticity, memory formation, and retention in neuronal cells [96,97]. Too much or too little mTOR activity could be fatal to neurons. A balance between activation of mTOR signaling and enhancement of autophagy needs to be accurately managed, which may offer exciting new avenues for the development of therapeutic strategies for PD. Though targeting of the mTOR pathway has shown neuroprotective actions in a variety of in vivo and in vitro PD models, the therapeutic potential of mTOR inhibitors (such as to enhance autophagy by inhibiting mTOR) may be limited because mTOR regulates multiple cellular functions. Additionally, mTOR inhibitors, such as rapamycin, may have some side effects in clinical trials [114,115]. For example, rapamycin has been applied in treating patients with lymphangioleiomyomatosis, leading to some rapamycin levels-associated side effects, like apthous ulcers, nausea, and diarrhea [114]. Thus, the dosage of mTOR inhibitors in clinical trials should be continuously modified during the treatment period [114]. Although several fundamental questions need to be further addressed before these novel mTOR-targeting reagents could be applied in clinical trials, the research field of mTOR is developing quickly and clinically relevant updates on mTOR modulators may arise soon.

## Abbreviations

ALR	Autophagic lysosome reformation
AMPK	AMP-activated protein kinase
CLEAR	Coordinated lysosomal expression and regulation
DEPTOR	DEP-domain containing mTOR-interacting protein
ER	Endoplasmic reticulum
FIP200	Focal adhesion kinase family interacting protein of 200 kD
FKBP	FK506-binding protein
FRAP	FKBP12-rapamycin complex-associated protein
GAP	GTPase-activating protein
GBA1	Glucosidase beta acid 1
IMPase	Inositol monophosphatase
IP3	Inositol 1,4,5-trisphosphate
IRS	Insulin receptor substrate
LRRK2	Leucine-rich repeat kinase 2
miR	MicroRNA
mLST8	Mammalian lethal with sec-13 protein 8
MPTP	1-methyl-4-phenyl-1,2,3,6 tetrahydropyridine
mSIN1	Mammalian stress-activated map kinase-interacting protein 1
mTOR	Mammalian targets of rapamycin
mTORC1	mTOR complex 1
mTORC2	mTOR complex 2
NRBF2	Nuclear receptor binding factor 2
PD	Parkinson’s disease
PDK1	3-Phosphoinositide dependent protein kinase-1
PI	Phosphatidylinositol
PINK1	PTEN-induced putative kinase 1
PI3K	Phosphatidylinositol 3-kinase
PI3P	Phosphatidylinositol 3-phosphate
PKC	Protein kinase C
PRAS40	Proline-rich Akt substrate of 40 kDa
Protor	Protein observed with Rictor
P70S6K	P70 ribosomal S6 kinase
Raptor	Regulatory associated protein of mammalian target of rapamycin
Rheb	Ras homolog enriched in brain
Rhes	Ras homolog enriched in striatum
Rictor	Rapamycin-insensitive companion of mTOR
SCARB2	Scavenger receptor class B member 2
SGK	Serum/glucocorticoid regulated kinase
SNpc	Substantia nigra pars compacta
TOR	Target of rapamycin
TSC	Tuberous sclerosis complex
ULK1	Unc51-like kinase 1
VPS34	Vacuolar protein sorting 34
VPS35	Vacuolar protein sorting 35
4EBP1	Eukaryotic initiation factor 4E (eIF4E) binding protein 1
6-OHDA	6-hydroxydopamine

## References

[1] Heitman J., Movva N.R., Hall M.N. (1991). Targets for cell cycle arrest by the immunosuppressant rapamycin in yeast. Science.

[2] Sabers C.J., Martin M.M., Brunn G.J., Williams J.M., Dumont F.J., Wiederrecht G., Abraham R.T. (1995). Isolation of a protein target of the FKBP12-rapamycin complex in mammalian cells. J. Biol. Chem..

[3] Lorenz M.C., Heitman J. (1995). TOR mutations confer rapamycin resistance by preventing interaction with FKBP12-rapamycin. J. Biol. Chem..

[4] Wullschleger S., Loewith R., Hall M.N. (2006). TOR signaling in growth and metabolism. Cell.

[5] Zheng X.F., Florentino D., Chen J., Crabtree G.R., Schreiber S.L. (1995). TOR kinase domains are required for two distinct functions, only one of which is inhibited by rapamycin. Cell.

[6] Peterson T.R., Laplante M., Thoreen C.C., Sancak Y., Kang S.A., Kuehl W.M., Gray N.S., Sabatini D.M. (2009). DEPTOR is an mTOR inhibitor frequently overexpressed in multiple myeloma cells and required for their survival. Cell.

[7] Hara K., Maruki Y., Long X., Yoshino K., Oshiro N., Hidayat S., Tokunaga C., Avruch J., Yonezawa K. (2002). Raptor, a binding partner of target of rapamycin (TOR), mediates TOR action. Cell.

[8] Kim D.H., Sarbassov D.D., Ali S.M., King J.E., Latek R.R., Erdjument-Bromage H., Tempst P., Sabatini D.M. (2002). mTOR interacts with raptor to form a nutrient-sensitive complex that signals to the cell growth machinery. Cell.

[9] Kim D.H., Sarbassov D.D., Ali S.M., Latek R.R., Guntur K.V., Erdjument-Bromage H., Tempst P., Sabatini D.M. (2003). GbetaL, a positive regulator of the rapamycin-sensitive pathway required for the nutrient-sensitive interaction between raptor and mTOR. Mol. Cell.

[10] Sancak Y., Thoreen C.C., Peterson T.R., Lindquist R.A., Kang S.A., Spooner E., Carr S.A., Sabatini D.M. (2007). PRAS40 is an insulin-regulated inhibitor of the mTORC1 protein kinase. Mol. Cell.

[11] Di Malta C., Siciliano D., Calcagni A., Monfregola J., Punzi S., Pastore N., Eastes A.N., Davis O., De Cegli R., Zampelli A. (2017). Transcriptional activation of RagD GTPase controls mTORC1 and promotes cancer growth. Science.

[12] Beirowski B., Wong K.M., Babetto E., Milbrandt J. (2017). mTORC1 promotes proliferation of immature Schwann cells and myelin growth of differentiated Schwann cells. Proc. Natl. Acad. Sci. USA.

[13] Chiarini F., Evangelisti C., McCubrey J.A., Martelli A.M. (2015). Current treatment strategies for inhibiting mTOR in cancer. Trends Pharmacol. Sci..

[14] Caron A., Richard D., Laplante M. (2015). The Roles of mTOR Complexes in Lipid Metabolism. Annu. Rev. Nutr..

[15] Wang B., Jie Z., Joo D., Ordureau A., Liu P., Gan W., Guo J., Zhang J., North B.J., Dai X. (2017). TRAF2 and OTUD7B govern a ubiquitin-dependent switch that regulates mTORC2 signalling. Nature.

[16] Sarbassov D.D., Ali S.M., Kim D.H., Guertin D.A., Latek R.R., Erdjument-Bromage H., Tempst P., Sabatini D.M. (2004). Rictor, a novel binding partner of mTOR, defines a rapamycin-insensitive and raptor-independent pathway that regulates the cytoskeleton. Curr. Biol..

[17] Frias M.A., Thoreen C.C., Jaffe J.D., Schroder W., Sculley T., Carr S.A., Sabatini D.M. (2006). mSin1 is necessary for Akt/PKB phosphorylation, and its isoforms define three distinct mTORC2s. Curr. Biol..

[18] Jacinto E., Loewith R., Schmidt A., Lin S., Ruegg M.A., Hall A., Hall M.N. (2004). Mammalian TOR complex 2 controls the actin cytoskeleton and is rapamycin insensitive. Nat. Cell Biol..

[19] Pearce L.R., Huang X., Boudeau J., Pawlowski R., Wullschleger S., Deak M., Ibrahim A.F., Gourlay R., Magnuson M.A., Alessi D.R. (2007). Identification of Protor as a novel Rictor-binding component of mTOR complex-2. Biochem. J..

[20] Mizunuma M., Neumann-Haefelin E., Moroz N., Li Y.J., Blackwell T.K. (2014). mTORC2-SGK-1 acts in two environmentally responsive pathways with opposing effects on longevity. Aging Cell.

[21] Chantaravisoot N., Wongkongkathep P., Loo J.A., Mischel P.S., Tamanoi F. (2015). Significance of filamin A in mTORC2 function in glioblastoma. Mol. Cancer.

[22] Chen B.W., Chen W., Liang H., Liu H., Liang C., Zhi X., Hu L.Q., Yu X.Z., Wei T., Ma T. (2015). Inhibition of mTORC2 Induces Cell-Cycle Arrest and Enhances the Cytotoxicity of Doxorubicin by Suppressing MDR1 Expression in HCC Cells. Mol. Cancer Ther..

[23] Albert V., Svensson K., Shimobayashi M., Colombi M., Munoz S., Jimenez V., Handschin C., Bosch F., Hall M.N. (2016). mTORC2 sustains thermogenesis via Akt-induced glucose uptake and glycolysis in brown adipose tissue. EMBO Mol. Med..

[24] Liu P., Gan W., Chin Y.R., Ogura K., Guo J., Zhang J., Wang B., Blenis J., Cantley L.C., Toker A. (2015). PtdIns(3,4,5)P3-Dependent Activation of the mTORC2 Kinase Complex. Cancer Discov..

[25] Sarbassov D.D., Guertin D.A., Ali S.M., Sabatini D.M. (2005). Phosphorylation and regulation of Akt/PKB by the rictor-mTOR complex. Science.

[26] Yang G., Murashige D.S., Humphrey S.J., James D.E. (2015). A Positive Feedback Loop between Akt and mTORC2 via SIN1 Phosphorylation. Cell Rep..

[27] Humphrey S.J., Yang G., Yang P., Fazakerley D.J., Stockli J., Yang J.Y., James D.E. (2013). Dynamic adipocyte phosphoproteome reveals that Akt directly regulates mTORC2. Cell Metab..

[28] Dibble C.C., Cantley L.C. (2015). Regulation of mTORC1 by PI3K signaling. Trends Cell Biol..

[29] Mabuchi S., Kuroda H., Takahashi R., Sasano T. (2015). The PI3K/AKT/mTOR pathway as a therapeutic target in ovarian cancer. Gynecol. Oncol..

[30] Inoki K., Li Y., Zhu T., Wu J., Guan K.L. (2002). TSC2 is phosphorylated and inhibited by Akt and suppresses mTOR signalling. Nat. Cell Biol..

[31] Brunn G.J., Hudson C.C., Sekulić A., Williams J.M., Hosoi H., Houghton P.J., Lawrence J.C., Abraham R.T. (1997). Phosphorylation of the Translational Repressor PHAS-I by the Mammalian Target of Rapamycin. Science.

[32] Burnett P.E., Barrow R.K., Cohen N.A., Snyder S.H., Sabatini D.M. (1998). RAFT1 phosphorylation of the translational regulators p70 S6 kinase and 4E-BP1. Proc. Natl. Acad. Sci. USA.

[33] Levy J.M.M., Towers C.G., Thorburn A. (2017). Targeting autophagy in cancer. Nat. Rev. Cancer.

[34] Arias E., Cuervo A.M. (2011). Chaperone-mediated autophagy in protein quality control. Curr. Opin. Cell Biol..

[35] Huber L.A., Teis D. (2016). Lysosomal signaling in control of degradation pathways. Curr. Opin. Cell Biol..

[36] Jung C.H., Jun C.B., Ro S.H., Kim Y.M., Otto N.M., Cao J., Kundu M., Kim D.H. (2009). ULK-Atg13-FIP200 complexes mediate mTOR signaling to the autophagy machinery. Mol. Biol. Cell.

[37] Hosokawa N., Sasaki T., Iemura S., Natsume T., Hara T., Mizushima N. (2009). Atg101, a novel mammalian autophagy protein interacting with Atg13. Autophagy.

[38] Hurley J.H., Young L.N. (2017). Mechanisms of Autophagy Initiation. Annu. Rev. Biochem..

[39] Kim J., Kundu M., Viollet B., Guan K.L. (2011). AMPK and mTOR regulate autophagy through direct phosphorylation of Ulk1. Nat. Cell Biol..

[40] Shang L., Chen S., Du F., Li S., Zhao L., Wang X. (2011). Nutrient starvation elicits an acute autophagic response mediated by Ulk1 dephosphorylation and its subsequent dissociation from AMPK. Proc. Natl. Acad. Sci. USA.

[41] Lin M.G., Hurley J.H. (2016). Structure and function of the ULK1 complex in autophagy. Curr. Opin. Cell Biol..

[42] Puente C., Hendrickson R.C., Jiang X. (2016). Nutrient-regulated Phosphorylation of ATG13 Inhibits Starvation-induced Autophagy. J. Biol. Chem..

[43] Egan D.F., Chun M.G., Vamos M., Zou H., Rong J., Miller C.J., Lou H.J., Raveendra-Panickar D., Yang C.C., Sheffler D.J. (2015). Small Molecule Inhibition of the Autophagy Kinase ULK1 and Identification of ULK1 Substrates. Mol. Cell.

[44] Backer J.M. (2016). The intricate regulation and complex functions of the Class III phosphoinositide 3-kinase Vps34. Biochem. J..

[45] Matsunaga K., Morita E., Saitoh T., Akira S., Ktistakis N.T., Izumi T., Noda T., Yoshimori T. (2010). Autophagy requires endoplasmic reticulum targeting of the PI3-kinase complex via Atg14L. J. Cell Biol..

[46] Tan X., Thapa N., Liao Y., Choi S., Anderson R.A. (2016). PtdIns(4,5)P2 signaling regulates ATG14 and autophagy. Proc. Natl. Acad. Sci. USA.

[47] Ma B., Cao W., Li W., Gao C., Qi Z., Zhao Y., Du J., Xue H., Peng J., Wen J. (2014). Dapper1 promotes autophagy by enhancing the Beclin1-Vps34-Atg14L complex formation. Cell Res..

[48] Byfield M.P., Murray J.T., Backer J.M. (2005). hVps34 is a nutrient-regulated lipid kinase required for activation of p70 S6 kinase. J. Biol. Chem..

[49] Nobukuni T., Joaquin M., Roccio M., Dann S.G., Kim S.Y., Gulati P., Byfield M.P., Backer J.M., Natt F., Bos J.L. (2005). Amino acids mediate mTOR/raptor signaling through activation of class 3 phosphatidylinositol 3OH-kinase. Proc. Natl. Acad. Sci. USA.

[50] Yuan H.-X., Russell R.C., Guan K.-L. (2014). Regulation of PIK3C3/VPS34 complexes by MTOR in nutrient stress-induced autophagy. Autophagy.

[51] Ohashi Y., Soler N., Garcia Ortegon M., Zhang L., Kirsten M.L., Perisic O., Masson G.R., Burke J.E., Jakobi A.J., Apostolakis A.A. (2016). Characterization of Atg38 and NRBF2, a fifth subunit of the autophagic Vps34/PIK3C3 complex. Autophagy.

[52] Ma X., Zhang S., He L., Rong Y., Brier L.W., Sun Q., Liu R., Fan W., Chen S., Yue Z. (2017). MTORC1-mediated NRBF2 phosphorylation functions as a switch for the class III PtdIns3K and autophagy. Autophagy.

[53] Araki Y., Ku W.C., Akioka M., May A.I., Hayashi Y., Arisaka F., Ishihama Y., Ohsumi Y. (2013). Atg38 is required for autophagy-specific phosphatidylinositol 3-kinase complex integrity. J. Cell Biol..

[54] Lu J., He L., Behrends C., Araki M., Araki K., Jun Wang Q., Catanzaro J.M., Friedman S.L., Zong W.X., Fiel M.I. (2014). NRBF2 regulates autophagy and prevents liver injury by modulating Atg14L-linked phosphatidylinositol-3 kinase III activity. Nat. Commun..

[55] Yang C., Cai C.Z., Song J.X., Tan J.Q., Durairajan S.S.K., Iyaswamy A., Wu M.Y., Chen L.L., Yue Z., Li M. (2017). NRBF2 is involved in the autophagic degradation process of APP-CTFs in Alzheimer disease models. Autophagy.

[56] Puertollano R., Ferguson S.M., Brugarolas J., Ballabio A. (2018). The complex relationship between TFEB transcription factor phosphorylation and subcellular localization. EMBO J..

[57] Sardiello M., Palmieri M., di Ronza A., Medina D.L., Valenza M., Gennarino V.A., Di Malta C., Donaudy F., Embrione V., Polishchuk R.S. (2009). A gene network regulating lysosomal biogenesis and function. Science.

[58] Martina J.A., Diab H.I., Lishu L., Jeong A.L., Patange S., Raben N., Puertollano R. (2014). The nutrient-responsive transcription factor TFE3 promotes autophagy, lysosomal biogenesis, and clearance of cellular debris. Sci. Signal..

[59] Raben N., Puertollano R. (2016). TFEB and TFE3: Linking Lysosomes to Cellular Adaptation to Stress. Annu. Rev. Cell Dev. Biol..

[60] Napolitano G., Esposito A., Choi H., Matarese M., Benedetti V., Di Malta C., Monfregola J., Medina D.L., Lippincott-Schwartz J., Ballabio A. (2018). mTOR-dependent phosphorylation controls TFEB nuclear export. Nat. Commun..

[61] Chen Y., Yu L. (2015). Scissors for autolysosome tubules. EMBO J..

[62] Munson M.J., Allen G.F., Toth R., Campbell D.G., Lucocq J.M., Ganley I.G. (2015). mTOR activates the VPS34-UVRAG complex to regulate autolysosomal tubulation and cell survival. EMBO J..

[63] Yu L., McPhee C.K., Zheng L., Mardones G.A., Rong Y., Peng J., Mi N., Zhao Y., Liu Z., Wan F. (2010). Termination of autophagy and reformation of lysosomes regulated by mTOR. Nature.

[64] Zhang J., Zhou W., Lin J., Wei P., Zhang Y., Jin P., Chen M., Man N., Wen L. (2016). Autophagic lysosomal reformation depends on mTOR reactivation in H2O2-induced autophagy. Int. J. Biochem. Cell Biol..

[65] Liu J., Liu W., Lu Y., Tian H., Duan C., Lu L., Gao G., Wu X., Wang X., Yang H. (2018). Piperlongumine restores the balance of autophagy and apoptosis by increasing BCL2 phosphorylation in rotenone-induced Parkinson disease models. Autophagy.

[66] Langston J.W., Ballard P., Tetrud J.W., Irwin I. (1983). Chronic Parkinsonism in humans due to a product of meperidine-analog synthesis. Science.

[67] Heikkila R.E., Manzino L., Cabbat F.S., Duvoisin R.C. (1984). Protection against the dopaminergic neurotoxicity of 1-methyl-4-phenyl-1,2,5,6-tetrahydropyridine by monoamine oxidase inhibitors. Nature.

[68] Recasens A., Dehay B., Bove J., Carballo-Carbajal I., Dovero S., Perez-Villalba A., Fernagut P.O., Blesa J., Parent A., Perier C. (2014). Lewy body extracts from Parkinson disease brains trigger alpha-synuclein pathology and neurodegeneration in mice and monkeys. Ann. Neurol..

[69] Wong Y.C., Krainc D. (2017). alpha-synuclein toxicity in neurodegeneration: Mechanism and therapeutic strategies. Nat. Med..

[70] Crews L., Spencer B., Desplats P., Patrick C., Paulino A., Rockenstein E., Hansen L., Adame A., Galasko D., Masliah E. (2010). Selective molecular alterations in the autophagy pathway in patients with Lewy body disease and in models of alpha-synucleinopathy. PLoS ONE.

[71] Gao S., Duan C., Gao G., Wang X., Yang H. (2015). Alpha-synuclein overexpression negatively regulates insulin receptor substrate 1 by activating mTORC1/S6K1 signaling. Int. J. Biochem. Cell Biol..

[72] Jiang T.F., Zhang Y.J., Zhou H.Y., Wang H.M., Tian L.P., Liu J., Ding J.Q., Chen S.D. (2013). Curcumin ameliorates the neurodegenerative pathology in A53T alpha-synuclein cell model of Parkinson’s disease through the downregulation of mTOR/p70S6K signaling and the recovery of macroautophagy. J. Neuroimmune Pharmacol..

[73] Malagelada C., Ryu E.J., Biswas S.C., Jackson-Lewis V., Greene L.A. (2006). RTP801 is elevated in Parkinson brain substantia nigral neurons and mediates death in cellular models of Parkinson’s disease by a mechanism involving mammalian target of rapamycin inactivation. J. Neurosci..

[74] Corradetti M.N., Inoki K., Guan K.L. (2005). The stress-inducted proteins RTP801 and RTP801L are negative regulators of the mammalian target of rapamycin pathway. J. Biol. Chem..

[75] Brugarolas J., Lei K., Hurley R.L., Manning B.D., Reiling J.H., Hafen E., Witters L.A., Ellisen L.W., Kaelin W.G. (2004). Regulation of mTOR function in response to hypoxia by REDD1 and the TSC1/TSC2 tumor suppressor complex. Genes Dev..

[76] Selvaraj S., Sun Y., Watt J.A., Wang S., Lei S., Birnbaumer L., Singh B.B. (2012). Neurotoxin-induced ER stress in mouse dopaminergic neurons involves downregulation of TRPC1 and inhibition of AKT/mTOR signaling. J. Clin. Investig..

[77] Xu Y., Liu C., Chen S., Ye Y., Guo M., Ren Q., Liu L., Zhang H., Xu C., Zhou Q. (2014). Activation of AMPK and inactivation of Akt result in suppression of mTOR-mediated S6K1 and 4E-BP1 pathways leading to neuronal cell death in in vitro models of Parkinson’s disease. Cell Signal..

[78] Rajput A.H. (2014). Factors predictive of the development of levodopa-induced dyskinesia and Wearing-Off in Parkinson’s disease. Mov. Disord..

[79] Huot P., Johnston T.H., Koprich J.B., Fox S.H., Brotchie J.M. (2013). The pharmacology of L-DOPA-induced dyskinesia in Parkinson’s disease. Pharmacol. Rev..

[80] Urs N.M., Bido S., Peterson S.M., Daigle T.L., Bass C.E., Gainetdinov R.R., Bezard E., Caron M.G. (2015). Targeting beta-arrestin2 in the treatment of l-DOPA-induced dyskinesia in Parkinson’s disease. Proc. Natl. Acad. Sci. USA.

[81] Martin-Flores N., Fernandez-Santiago R., Antonelli F., Cerquera C., Moreno V., Marti M.J., Ezquerra M., Malagelada C. (2018). MTOR Pathway-Based Discovery of Genetic Susceptibility to L-DOPA-Induced Dyskinesia in Parkinson’s Disease Patients. Mol. Neurobiol..

[82] Santini E., Heiman M., Greengard P., Valjent E., Fisone G. (2009). Inhibition of mTOR signaling in Parkinson’s disease prevents L-DOPA-induced dyskinesia. Sci. Signal..

[83] Subramaniam S., Napolitano F., Mealer R.G., Kim S., Errico F., Barrow R., Shahani N., Tyagi R., Snyder S.H., Usiello A. (2011). Rhes, a striatal-enriched small G protein, mediates mTOR signaling and L-DOPA-induced dyskinesia. Nat. Neurosci..

[84] Brugnoli A., Napolitano F., Usiello A., Morari M. (2016). Genetic deletion of Rhes or pharmacological blockade of mTORC1 prevent striato-nigral neurons activation in levodopa-induced dyskinesia. Neurobiol. Dis..

[85] Moors T.E., Hoozemans J.J., Ingrassia A., Beccari T., Parnetti L., Chartier-Harlin M.C., van de Berg W.D. (2017). Therapeutic potential of autophagy-enhancing agents in Parkinson’s disease. Mol. Neurodegener..

[86] Verstraeten A., Theuns J., Van Broeckhoven C. (2015). Progress in unraveling the genetic etiology of Parkinson disease in a genomic era. Trends Genet..

[87] Przedborski S. (2017). The two-century journey of Parkinson disease research. Nat. Rev. Neurosci..

[88] Gan-Or Z., Dion P.A., Rouleau G.A. (2015). Genetic perspective on the role of the autophagy-lysosome pathway in Parkinson disease. Autophagy.

[89] Rocha E.M., Smith G.A., Park E., Cao H., Brown E., Hallett P., Isacson O. (2015). Progressive decline of glucocerebrosidase in aging and Parkinson’s disease. Ann. Clin. Transl. Neurol..

[90] Decressac M., Mattsson B., Weikop P., Lundblad M., Jakobsson J., Björklund A. (2013). TFEB-mediated autophagy rescues midbrain dopamine neurons from α-synuclein toxicity. Proc. Natl. Acad. Sci. USA.

[91] Boland B., Yu W.H., Corti O., Mollereau B., Henriques A., Bezard E., Pastores G.M., Rubinsztein D.C., Nixon R.A., Duchen M.R. (2018). Promoting the clearance of neurotoxic proteins in neurodegenerative disorders of ageing. Nat. Rev. Drug Discov..

[92] Dehay B., Bove J., Rodriguez-Muela N., Perier C., Recasens A., Boya P., Vila M. (2010). Pathogenic lysosomal depletion in Parkinson’s disease. J. Neurosci..

[93] Malagelada C., Jin Z.H., Jackson-Lewis V., Przedborski S., Greene L.A. (2010). Rapamycin protects against neuron death in in vitro and in vivo models of Parkinson’s disease. J. Neurosci..

[94] Bove J., Martinez-Vicente M., Vila M. (2011). Fighting neurodegeneration with rapamycin: Mechanistic insights. Nat. Rev. Neurosci..

[95] Liu J., Chen M., Wang X., Wang Y., Duan C., Gao G., Lu L., Wu X., Wang X., Yang H. (2016). Piperine induces autophagy by enhancing protein phosphotase 2A activity in a rotenone-induced Parkinson’s disease model. Oncotarget.

[96] Bekinschtein P., Katche C., Slipczuk L.N., Igaz L.M., Cammarota M., Izquierdo I., Medina J.H. (2007). mTOR signaling in the hippocampus is necessary for memory formation. Neurobiol. Learn. Mem..

[97] Hoeffer C.A., Klann E. (2010). mTOR signaling: At the crossroads of plasticity, memory and disease. Trends Neurosci..

[98] Sarkar S., Floto R.A., Berger Z., Imarisio S., Cordenier A., Pasco M., Cook L.J., Rubinsztein D.C. (2005). Lithium induces autophagy by inhibiting inositol monophosphatase. J. Cell Biol..

[99] Sarkar S., Davies J.E., Huang Z., Tunnacliffe A., Rubinsztein D.C. (2007). Trehalose, a novel mTOR-independent autophagy enhancer, accelerates the clearance of mutant huntingtin and alpha-synuclein. J. Biol. Chem..

[100] Song J.X., Lu J.H., Liu L.F., Chen L.L., Durairajan S.S., Yue Z., Zhang H.Q., Li M. (2014). HMGB1 is involved in autophagy inhibition caused by SNCA/alpha-synuclein overexpression: A process modulated by the natural autophagy inducer corynoxine B. Autophagy.

[101] Song J.X., Sun Y.R., Peluso I., Zeng Y., Yu X., Lu J.H., Xu Z., Wang M.Z., Liu L.F., Huang Y.Y. (2016). A novel curcumin analog binds to and activates TFEB in vitro and in vivo independent of MTOR inhibition. Autophagy.

[102] Sarkar S., Ravikumar B., Floto R.A., Rubinsztein D.C. (2009). Rapamycin and mTOR-independent autophagy inducers ameliorate toxicity of polyglutamine-expanded huntingtin and related proteinopathies. Cell Death Differ..

[103] Dauer W., Przedborski S. (2003). Parkinson’s disease: Mechanisms and models. Neuron.

[104] Domanskyi A., Geissler C., Vinnikov I.A., Alter H., Schober A., Vogt M.A., Gass P., Parlato R., Schutz G. (2011). Pten ablation in adult dopaminergic neurons is neuroprotective in Parkinson’s disease models. FASEB J..

[105] Long X., Lin Y., Ortiz-Vega S., Yonezawa K., Avruch J. (2005). Rheb binds and regulates the mTOR kinase. Curr. Biol..

[106] Inoki K., Li Y., Xu T., Guan K.L. (2003). Rheb GTPase is a direct target of TSC2 GAP activity and regulates mTOR signaling. Genes Dev..

[107] Aspuria P.J., Tamanoi F. (2004). The Rheb family of GTP-binding proteins. Cell Signal..

[108] Potter C.J., Pedraza L.G., Xu T. (2002). Akt regulates growth by directly phosphorylating Tsc2. Nat. Cell Biol..

[109] Kim S.R., Chen X., Oo T.F., Kareva T., Yarygina O., Wang C., During M., Kholodilov N., Burke R.E. (2011). Dopaminergic pathway reconstruction by Akt/Rheb-induced axon regeneration. Ann. Neurol..

[110] Iwakawa H.O., Tomari Y. (2015). The Functions of MicroRNAs: mRNA Decay and Translational Repression. Trends Cell Biol..

[111] Wilczynska A., Bushell M. (2015). The complexity of miRNA-mediated repression. Cell Death Differ..

[112] Ma L., Wei L., Wu F., Hu Z., Liu Z., Yuan W. (2013). Advances with microRNAs in Parkinson’s disease research. Drug Des. Dev.Ther..

[113] Fragkouli A., Doxakis E. (2014). miR-7 and miR-153 protect neurons against MPP(+)-induced cell death via upregulation of mTOR pathway. Front. Cell. Neurosci..

[114] Bee J., Fuller S., Miller S., Johnson S.R. (2018). Lung function response and side effects to rapamycin for lymphangioleiomyomatosis: A prospective national cohort study. Thorax.

[115] Lamming D.W., Ye L., Sabatini D.M., Baur J.A. (2013). Rapalogs and mTOR inhibitors as anti-aging therapeutics. J. Clin. Investig..

